# CT Simplified Radiomic Approach to Assess the Metastatic Ductal Adenocarcinoma of the Pancreas

**DOI:** 10.3390/cancers13081843

**Published:** 2021-04-13

**Authors:** Mirko D’Onofrio, Riccardo De Robertis, Gregorio Aluffi, Camilla Cadore, Alessandro Beleù, Nicolò Cardobi, Giuseppe Malleo, Erminia Manfrin, Claudio Bassi

**Affiliations:** 1 Department of Radiology, G.B. Rossi Hospital, University of Verona, 37129 Verona, Italy; gregorio.aluffi@studenti.univr.it (G.A.); camilla.cadore@studenti.univr.it (C.C.); abeleu@ospedalepederzoli.it (A.B.); 2 Department of Radiology, Ospedale Civile Maggiore, Azienda Ospedaliera Universitaria Integrata Verona, 37126 Verona, Italy; riccardo.derobertislombardi@aovr.veneto.it (R.D.R.); nicolo.cardobi@aovr.veneto.it (N.C.); 3 Unit of General and Pancreatic Surgery, The Pancreas Institute, Policlinico GB Rossi, University of Verona, 37129 Verona, Italy; giuseppe.malleo@univr.it (G.M.); claudio.bassi@univr.it (C.B.); 4 Department of Pathology, G.B. Rossi Hospital, University of Verona, 37129 Verona, Italy; erminia.manfrin@univr.it

**Keywords:** radiomics, pancreatic adenocarcinoma, pancreatic cancer, CT, metastasis

## Abstract

**Simple Summary:**

In the diagnostic and therapeutic path of the ductal adenocarcinoma of the pancreas, an early detection of the lesion, a correct staging, and the consequent indication or not of the resectability are key elements. The purpose of this study is to analyze qualitative and quantitative computed tomography characteristics, for the purpose of staging integration and prognostic stratification. This could be useful both to avoid neglecting potentially resectable tumors and, above all, to avoid surgically treating tumors that are not properly staged, delaying or precluding the efficacy of the pharmacological therapeutic approach.

**Abstract:**

The aim of this study was to perform a simplified radiomic analysis of pancreatic ductal adenocarcinoma based on qualitative and quantitative tumor features and to compare the results between metastatic and non-metastatic patients. A search of our radiological, surgical, and pathological databases identified 1218 patients with a newly diagnosed pancreatic ductal adenocarcinoma who were referred to our Institution between January 2014 and December 2018. Computed Tomography (CT) examinations were reviewed analyzing qualitative and quantitative features. Two hundred eighty-eight patients fulfilled the inclusion criteria and were included in this study. Overall, metastases were present at diagnosis in 86/288 patients, while no metastases were identified in 202/288 patients. Ill-defined margins and a hypodense appearance on portal-phase images were significantly more common among patients with metastases compared to non-metastatic patients (*p* < 0.05). Metastatic tumors showed a significantly larger size and significantly lower arterial index, perfusion index, and permeability index compared to non-metastatic tumors (*p* < 0.05). In the management of pancreatic ductal adenocarcinoma, early detection and correct staging are key elements. The study of computerized tomography characteristics of pancreatic ductal adenocarcinoma showed substantial differences, both qualitative and quantitative, between metastatic and non-metastatic disease.

## 1. Introduction

Pancreatic ductal adenocarcinoma (PDAC) accounts for more than 80% of pancreatic neoplasms and represents the fourth cause of cancer-related death worldwide [1]. Despite advances in early diagnosis and multimodal treatments, the prognosis for patients with PDAC remains poor, with a 5-year survival rate that lies around 8% [2]. Surgical resection, which is feasible in 20% of patients, is the only potentially curative treatment for PDAC [3]. However, within resectable tumors, the expected median survival after resection lies around 23 months when negative surgical margins are achieved and 11 months with positive resection margins [4]. Furthermore, several patients experience early tumor recurrence after surgery, even at an early disease stage. Previous studies demonstrated that PDAC is a heterogeneous disease: multiple aberrant signaling pathways are involved in tumor development and growth, leading to several subsets of tumors with different clinical behaviors [5,6,7,8]. In this scenario, a multimodal strategy tailored on inner tumor characteristics is a key factor for effective tumor treatment [9,10,11]. Preoperative imaging for PDAC is commonly based on computed tomography (CT), which has a pivotal role in ruling out distant metastases and evaluating the involvement of peripancreatic vessels [6]. In recent years, the research in the field of radiology strongly focused on the extraction of inner tumor features by the high throughput analysis of biomedical images through the process known as radiomics. Previous reports have shown that a radiomic approach to PDAC may show promising results [12,13,14], but the application of such analysis to clinical practice is still very limited, due to its complexity. However, a simplified approach to radiomics may be feasible: for example, Choi et al. [15] reported a correlation between non-complex features, as tumor margins, and DPC4 expression in PDAC patients. Since metastases are the most relevant negative prognostic factor in pancreatic ductal adenocarcinoma, the availability of biomarkers that could identify patients that will likely develop metastases would be useful to stratify patients’ prognosis and to optimize their therapeutic management [16]. In particular, a simple imaging-based evaluation has been tested in the present paper to identify more aggressive ductal adenocarcinoma, in terms of the propensity of the lesion to metastasize, analyzing the characteristics of the primary tumor. An evaluation without competed assistance analysis has been purposed to conform with everyday clinical practice, but adding parameters calculated to suggest tumor aggressiveness. The aim is to be more precise in identifying the aggressive tumors tending to metastasize even without visible metastases at the time of diagnosis, asking for proper genetic profiling in these cases to have a confirmation and thus to improve the management of each single case.

The aim of this study was to perform a simplified radiomic analysis of PDAC based on qualitative and quantitative tumor features and to compare the results between metastatic and non-metastatic tumors.

## 2. Material and Methods

This retrospective study was approved by our Institutional Review Board, and the requirement for informed consent was waived.

A search of our radiological, surgical, and pathological databases identified 1218 patients with a newly diagnosed PDAC that referred to our Institution between January 2014 and December 2018.

**Figure 1:**
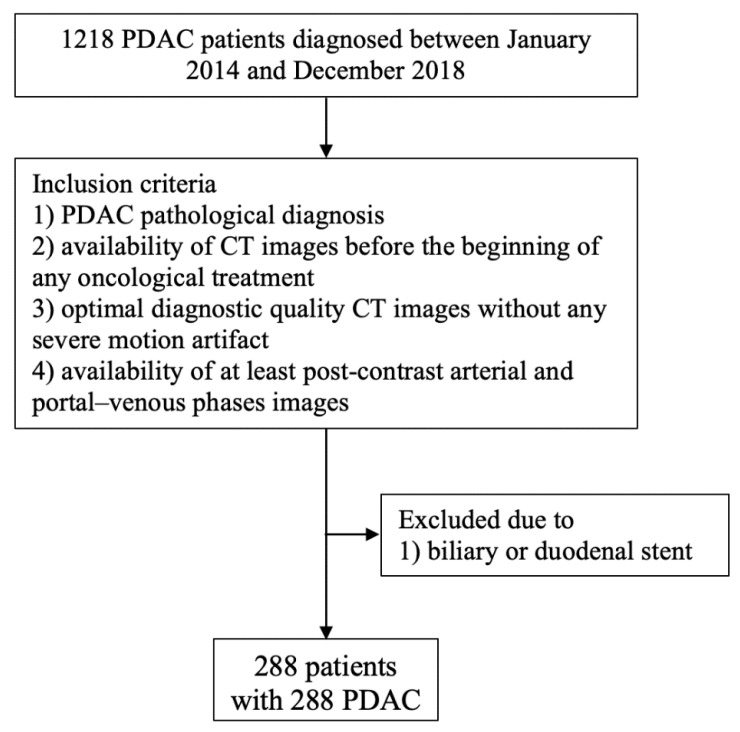
Flow chart showing patient selection criteria.

The inclusion criteria of this study were: (1) a pathological diagnosis of PDAC by means of fine-needle aspiration (FNA) or pathological analysis of the surgical specimen; (2) the availability of CT images performed before any treatment, comprising at least arterial- and portal-phase images without any severe artifact. Procedures were performed within one month. Patients having a biliary or a duodenal stent were excluded. A flow chart showing patient selection is presented in Figure 1.

CT examinations were performed with a 64-row equipment (Brilliance 64, Philips, Eindhoven, The Netherlands) before and after the administration of a weight-based amount of iodine contrast medium at the dose of 1.5 mL/kg. The following parameters were applied for all scans: reconstruction thickness 2 mm, pitch 1, kV 120, and mAs 125–250. All examinations were acquired by using a standard CT protocol for pancreatic study. The timing for post-contrast scans was based on a bolus tracking technique (15 s after the aortic peak enhancement for arterial phase images, 60–70 s for venous phase images, and 180–300 s for delayed phase images).

The rationale of our choice of parameters comes from the infiltration growth patterns and tumoral vascularization changes in more aggressive tumors.

A radiologist with 20 years of experience in pancreatic diseases, blinded to the pathological results, retrospectively reviewed each examination.

The following qualitative features were analyzed: tumor location (head or body/tail); tumor margin characteristics (well/ill defined, assessed on axial image by the presence of spiculation/infiltration at the tumor margins); presence or absence of arterial and venous infiltration; presence or absence of metastases; tumor appearance on post-contrast images compared to the adjacent pancreatic parenchyma (iso-, hypo-o hyperdense).

The following quantitative features were analyzed: tumor size, expressed as the maximum diameter of the lesion; tumor density, expressed as Hounsfield units (HU); the arterial index, defined as the ratio between the tumor and aortic density on arterial phase images; the perfusion index, defined as the ratio between the sum of arterial- and portal-phase tumor density and the aortic density on arterial phase images; and the permeability index, defined as the ratio between the difference of arterial and portal-phase density and the aortic density on arterial phase images. The region of interest (ROI) was manually drawn by a radiologist with 20 years of experience in pancreatic disease.

Patients were grouped based on the presence or absence of metastases. Metastases were confirmed by means of fine needle aspiration (FNA), surgical resection, or unequivocal imaging appearance on basal and follow-up examinations. The ANOVA test was used for parametric group comparisons, while a Kruskal–Wallis test was used for non-parametric features. Continuous variables with a normal distribution are presented as mean ± standard deviation, while variables with a non-normal distribution are presented as median [interquartile range]. Discrete variables are presented as relative percentage. The level of significance was set at *p* < 0.05 with a confidence interval of 95%. A statistical analysis was performed by using SPSS Statistic, version 19 (IBM, Chicago, IL, USA).

## 3. Results

Two hundred eighty-eight patients (148 men, 140 women; mean age 68.4 years; age range 41–90 years) fulfilled the inclusion criteria and were included in this study. Arterial- and portal-phase images were available for all patients; non-contrast CT scan was available for 258 patients (89.5%), and delayed phase images were available for 71 patients (24.6%).

Qualitative features are presented in Table 1.

Overall, the tumor was located in the pancreatic head (right pancreas) in 153 patients (53.13%) and in the pancreatic body–tail (left pancreas) in 135 patients. In 86 (29.87%) cases, distant metastases (liver /lung) were evident at the time of diagnosis. Tumor margins resulted well-defined in 138 (47.92%) cases and ill-defined in the remaining 150 (52.08%). Arterial invasion was detected in 153 (53.13%) cases. Venous invasion was observed in 181 (62.85%) patients.

Tumor appearance on post-contrast images showed that in the arterial phase 272 (94.44%) lesions appeared hypodense, while only 16 (5.56%) were isodense; in the portal-venous phase, 188 (65.28%) lesions appeared hypodense, 84 (29.17%) isodense, and only 16 (5.55%) hyperdense; in the delayed phase, available in 71 patients, 20 (28.17%) lesions appeared isodense, 27 (38%) hypodense, and 24 (33.8%) hyperdense.

The qualitative analysis in metastatic and non-metastatic samples is summarized in Table 1.

**Figure 2:**
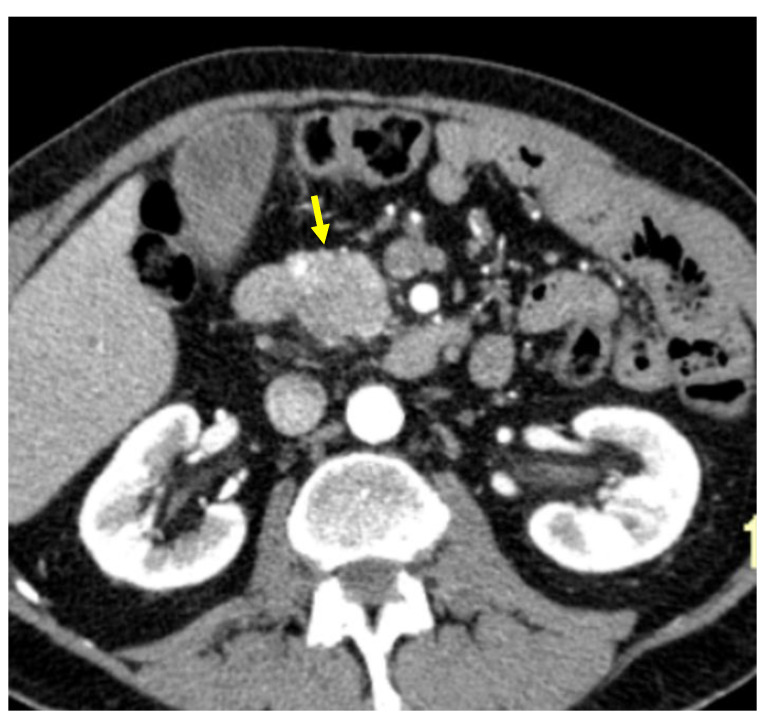
Pancreatic lesion with well-defined margins (arrow) on CT examination in pancreatic contrast phase.

**Figure 3:**
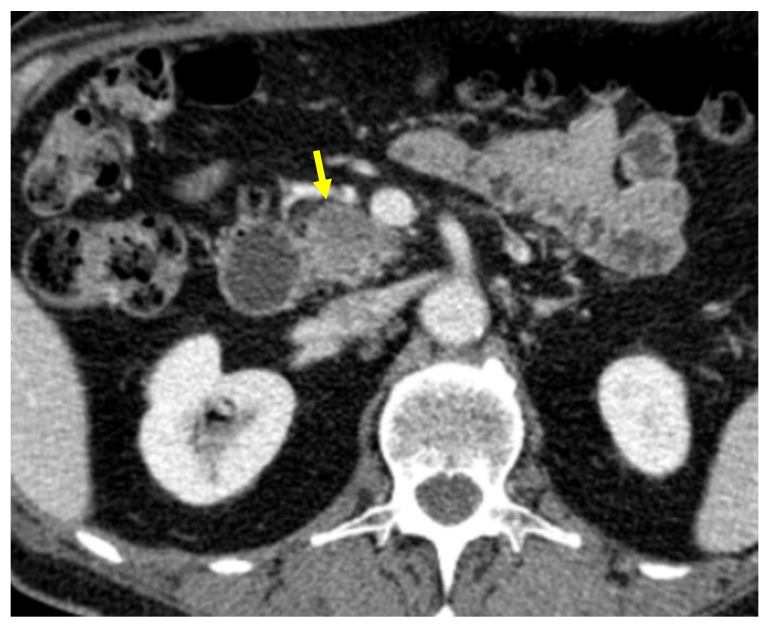
Pancreatic lesion with well-defined margins (arrow) on CT examination in portal-venous contrast phase.

**Figure 4:**
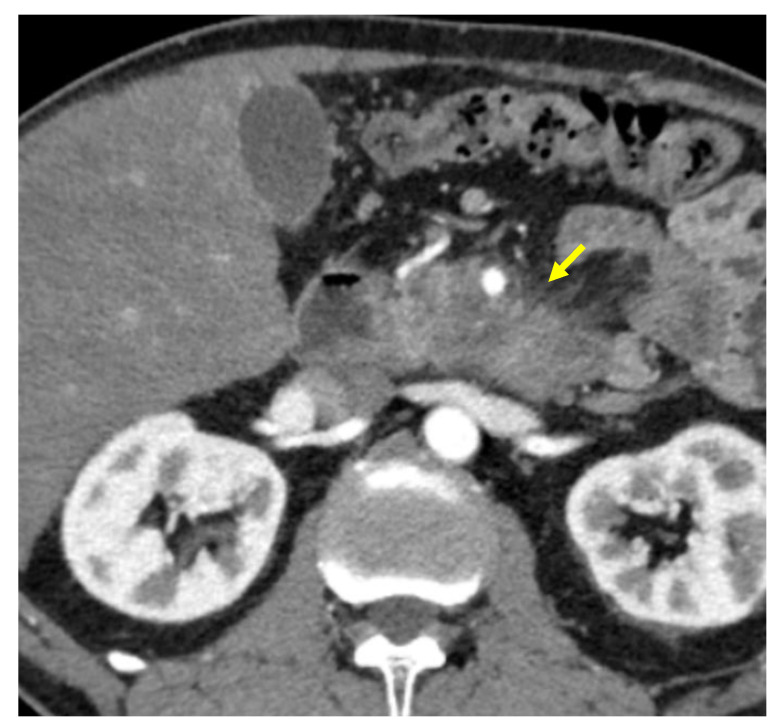
Pancreatic lesion with ill-defined margins (arrow) on CT examination in pancreatic contrast phase.

**Figure 5:**
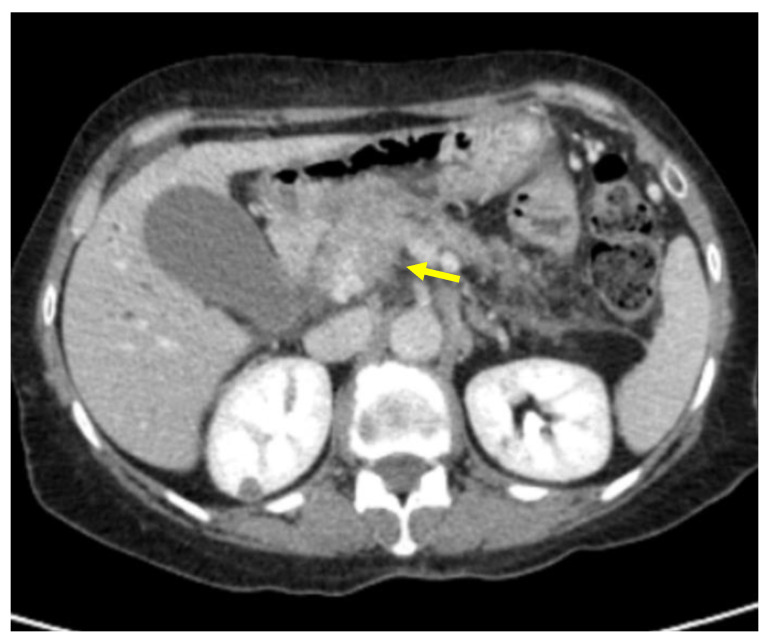
Pancreatic lesion with ill-defined margins (arrow) on CT examination in portal-venous contrast phase.

Tumor margins showed a significant difference in the two groups, appearing well-defined in only 6 (6.98%) metastatic patients and ill-defined in the remaining 80 (93.02%); in the non-metastatic group, the margins appeared well-defined in 132 (65.35%) and ill-defined in 70 (34.65%) tumors (Figure 2, Figure 3, Figure 4 and Figure 5).

Arterial invasion was detected in 74 (86.04%) metastatic patients and in 79 (39.11%) non-metastatic patients. Venous invasion was observed in 83 (96.51%) metastatic patients and in 98 (48.51%) non-metastatic patients.

Visual lesion assessment in the portal-venous phase showed a significant statistical difference in the two groups, appearing hypodense, respectively, in 80 (93.02%) metastatic patients and 108 (53.46%) non-metastatic patients.

Quantitative features are presented in Table 2.

The average major diameter measured 31.92 mm, with a maximum of 67 mm and minimum of 14 mm. The mean tumor density was 34 HU on pre-contrast scans (range, 19–45 HU), 59.3 HU on arterial-phase images (range, 23–119 HU), 75.2 HU on portal-phase images (range, 32–128 HU), and 82.1 HU on delayed-phase images (range, 33–125 HU).

Regarding the indices: the mean arterial index was 0.22 (range, 0.09–0.53); the mean perfusion index was 0.49 (range, 0.19–1.08); the mean permeability index was 0.06 (range, 0.01–0.4).

The quantitative analysis in metastatic and non-metastatic samples is summarized in Table 2**.**

The size showed a significant difference between the two groups: in the metastatic one, the average major diameter measured 40.36 mm (range, 17–67 mm), and 28.31 mm (range, 14–55 mm) in the non-metastatic.

Metastatic tumors presented lower arterial, perfusion, and permeability indices: the mean arterial index was 0.19 (range, 0.09–0.385) in metastatic patients and 0.23 (range, 0.1–0.53) in non-metastatic patients; the mean perfusion index was 0.45 (range, 0.2–0.24) in metastatic patients and 0.52 (range, 0.79–1.08) in non-metastatic patients; the mean permeability index was 0.045 (range, 0.006–0.132) in metastatic patients and 0.067 (range, 0.007–0.4) in non-metastatic patients.

In summary, ill-defined margins, hypodense appearance in the portal-phase, larger size, and lower arterial, perfusion, and permeability indices were significantly more common among patients with metastases compared to non-metastatic patients (*p*-value < 0.05).

## 4. Discussion

About 40% of patients with PDAC present distant metastases at diagnosis. Since metastases are the most relevant negative prognostic factor, even for technically resectable lesions, the availability of biomarkers that could identify patients that will likely develop metastases would be useful to stratify patients’ prognosis and to optimize their therapeutic management. In particular, a simple imaging-based evaluation has been tested in the present paper to identify more aggressive ductal adenocarcinoma in terms of propensity of the lesion to metastasize, analyzing the characteristics of the primary tumor. Evaluation without competed assistance analysis has been purposed, to conform with everyday clinical practice, but adding parameters calculated to suggest tumor aggressiveness.

Previous studies reported that radiomics, and radiogenomics, have a potential usefulness in tumor grading prediction, so in the identification of patients at high risk of early tumor recurrence after surgery as well as of distant metastases, and in the evaluation of tumor response to treatment [17,18]. A major limitation of radiomics is that the complex analysis that generates radiomic-based biomarkers is largely unapplicable to clinical practice. A simplified approach would therefore be useful for everyday practice. In this study, we retrospectively analyzed several features in a heterogeneous cohort composed by metastatic, locally advanced, and resectable PDAC patients, aiming to evaluate differences between metastatic and non-metastatic patients in order to provide possible biomarkers predictive of metastatic spread. Six of these parameters supported the hypothesis. Regarding the qualitative analysis, we found that tumors with ill-defined margins were significantly more common among metastatic patients: this finding is in line with the study by Choi et al., which observed that PDACs with well-defined margins were significantly associated with DPC4 expression, a tumor-suppressor gene known to have a pivotal role in widespread metastases [15]. Moreover, hypodense tumors on portal-phase images were significantly more frequent among patients with metastatic spread; this finding was corroborated by the analysis of the density values, that showed lower attenuation values in metastatic patients. Regarding the evaluation of the three indices (arterial, perfusion, and permeability), it was observed that metastatic patients presented with significantly lower mean values than non-metastatic ones. This finding is of great interest, considering that these indices are easily calculated and not particularly operator-dependent, since the main limit is represented by the ROI selection of the pancreatic lesion.

Furthermore, the two groups showed a significant difference in size, with the average lesion’s maximum diameter greater than 12 mm in the metastatic sample, in accordance with the previous literature [19,20].

Vascular invasion, both arterial and venous, was significantly more prevalent in metastatic patients, as expected.

We speculate that the CT findings of the tested parameters may indicate an aggressive subset of PDAC that has a high likelihood of metastasizing. The rationale of our choice of parameters comes from the infiltration growth patterns and tumoral vascularization changes in more aggressive tumors. We argue that irregular margins could reflect more infiltrative growth patterns, and hypoperfusion could be related to intralesional necrosis.

We suggest that these intrinsic tumor features, some of which have not been previously evaluated in PDAC patients (arterial, perfusion, and permeability indices), may potentially be assimilated into the conventional radiological assessment in order to better evaluate patients who may more likely benefit from surgical resection, and to differentiate them from patients at high risk of metastasis in which other forms of treatment or further investigation with genetic profiling should be considered.

The main advantage of the tested parameters is their practicality, making them standardizable and more applicable in clinical practice than other, more sophisticated analyses. Indeed, all the analyses were performed on a standard workstation with no need of imaging reconstruction or use of external software.

Nevertheless, the promising results of previous radiomics research, especially of texture analysis [12,13,17,18], show that the different data analysis might be complementary and integrated in a complex staging system. Radiomics remains a central research field for pancreatic cancer [21,22]. However, simple CT qualitative and quantitative parameters could be useful, complementary, and integrated in a pancreatic tumor imaging data evaluation form.

The main limitations of this study are its retrospective nature and an unbalanced sampling, with a majority of non–metastatic patients at diagnosis. The study findings have not been validated in an independent dataset, and a consecutive prospective validation study is therefore required to confirm the present results.

The fact that the study population was heterogeneously composed was a deliberate choice, the aim being the lesion assessment and its association with the presence of metastases, regardless of other aspects such as vascular invasion or grading. Moreover, the analysis of CT parameters was performed by a single expert radiologist, so that interobserver agreement was not assessable.

However, the impact of these factors on our results cannot be excluded. Therefore, further prospective studies, possibly on a larger and more homogeneous population, will be needed to confirm what has been observed.

## 5. Conclusions

In the management of pancreatic ductal adenocarcinoma, early detection and correct staging are key elements.The study of computerized tomography characteristics of pancreatic ductal adenocarcinoma showed substantial differences, both qualitative and quantitative, between metastatic and non-metastatic disease.

## Figures and Tables

**Table 1:** Qualitative descriptors in metastatic (M+) and non-metastatic (M−) patients.

	NUMBER		PERCENTAGE		TOT.ANALYSIS	
	M+	M−	M+	M−	M+	M−
**LOCATION**						
head	36	117	41.86%	57.92%	86/86	202/202
body	50	85	58.14%	42.08%		
**MARGINS**						
well-defined	6	132	6.98%	65.35%	86/86	202/202
ill-defined	80	70	93.02%	34.65%		
**VASCULAR INFILTRATION**						
arterial	74	79	86.04%	39.11%	86/86	202/202
venous	83	98	96.51%	48.51%		
**ARTERIAL DENSITY**						
iso	1	15	1.16%	7.43%		
hypo	85	187	98.84%	92.57%	86/86	202/202
hyper	0	0	0%	0%		
**VENOUS DENSITY**						
iso	6	78	6.98%	38.81%		
hypo	80	108	93.02%	53.46%	86/86	202/202
hyper	0	16	0%	7.92%		
**DELAYED DENSITY**						
iso	5	15	20.83%	31.91%		
hypo	17	10	70.83%	21.28%	24/86	47/202
hyper	2	22	8.34%	46.81%		

**Table 2:** Quantitative descriptors in metastatic (M+) and non-metastatic (M−) patients.

	MEAN		RANGE		TOT. ANALYSIS	
	M+	M−	M+	M−	M+	M−
**MAJOR** **DIAMETER (mm)**	40.36	28.31	17–67	14–55	86/86	201/202
**DENSITY (HU)**						
basal	33.30	34.33	19–43	20–45	74/86	184/202
arterial	52.02	62.46	23–86	30–119	86/86	202/202
portal-venous	63.91	79.97	32–95	40–128	86/86	202/202
delayed	71.60	87.51	33–102	45–125	25/86	49/202
**ARTERIAL**						
**INDEX**	0.19201615	0.22798981	0.094147583–0.385	0.101973684–0.533333333	86/86	202/202
**PERFUSION**						
**INDEX**	0.44938252	0.51997185	0.198473282–0.79	0.243243243–1.078787879	85/86	199/202
**PERMEABILITY**						
**INDEX**	0.044859953	0.06674615	0.006369427–0.13229572	0.00724638–0.4	85/86	199/202

## Data Availability

Data Availability contacting the corresponding Authors, University of Verona.
